# SAPK10-Mediated Phosphorylation on WRKY72 Releases Its Suppression on Jasmonic Acid Biosynthesis and Bacterial Blight Resistance

**DOI:** 10.1016/j.isci.2019.06.009

**Published:** 2019-06-11

**Authors:** Yuxuan Hou, Yifeng Wang, Liqun Tang, Xiaohong Tong, Ling Wang, Lianmeng Liu, Shiwen Huang, Jian Zhang

**Affiliations:** 1 State Key Lab of Rice Biology, China National Rice Research Institute, Hangzhou 311400, China

**Keywords:** Biochemistry, Cell Biology, Genetics, Microbiology, Molecular Biology, Plant Biology

## Abstract

Bacterial blight caused by the infection of *Xanthomonas oryzae* pv. *oryzae* (*Xoo*) is a devastating disease that severely challenges the yield of rice. Here, we report the identification of a “SAPK10-WRKY72-AOS1” module, through which *Xoo* infection stimulates the suppression of jasmonic acid (JA) biosynthesis to cause *Xoo* susceptibility. WRKY72 directly binds to the W-box in the promoter of JA biosynthesis gene *AOS1* and represses its transcription by inducing DNA hypermethylation on the target site, which finally led to lower endogenous JA level and higher *Xoo* susceptibility. Abscisic acid (ABA)-inducible SnRK2-type kinase SAPK10 phosphorylates WRKY72 at Thr 129. The SAPK10-mediated phosphorylation impairs the DNA-binding ability of WRKY72 and releases its suppression on *AOS1* and JA biosynthesis. Our work highlights a module of how pathogen stimuli lead to plant susceptibility, as well as a potential pathway for ABA-JA interplay with post-translational modification and epigenetic regulation mechanism involved.

## Highlights

• WRKY72 negatively regulates rice resistance to *Xoo* infection and JA synthesis
• SAPK10 phosphorylates WRKY72 at Thr 129 to impair its DNA binding on *AOS1*
• WRKY72 directly represses *AOS1* transcription to attenuate JA synthesis
• WRKY72 recruits hyper DNA methylation on *AOS1* promoter

## Graphical Abstract

**Figure:**
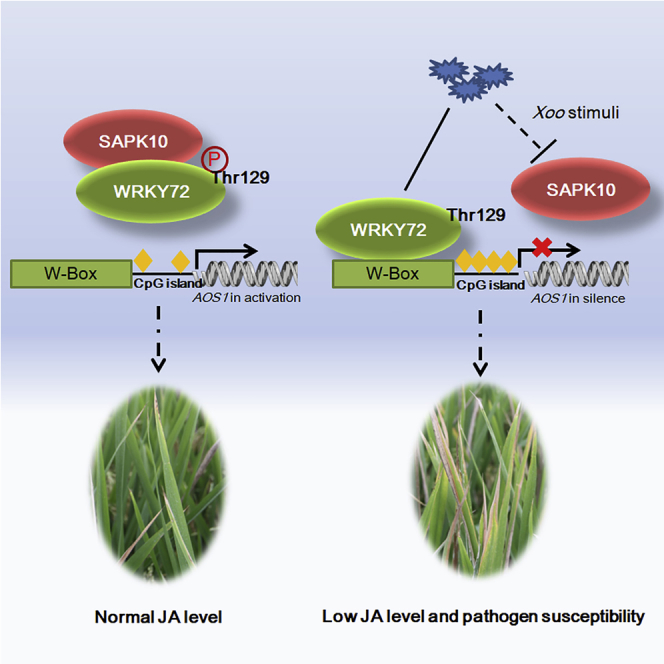

## Introduction

The plant innate immune system is considered to contain two interconnected layers termed PTI (pathogen-associated molecular patterns-triggered immunity) and ETI (effector-triggered immunity) (Jones and Dangl, 2006). Once plant intercepts pathogen-associated molecular patterns (PAMPs) such as chitin and flagellin, it activates downstream defense signaling to provide the first layer (Jones and Dangl, 2006, Saijo et al., 2018). Some virulent pathogens secrete effector proteins to suppress PTI. To fight back, plant resistance (R) proteins trigger ETI that provokes highly efficient defense responses upon effectors (Jones and Dangl, 2006, Peng et al., 2017). PTI and ETI usually result in massive transcriptional reprogramming of defense genes, which indicates the existence of a complex regulatory circuitry composed of transcriptional activators and repressors (Agarwal and Chikara, 2011, Madhunita and Ralf, 2014).

The WRKY family proteins are plant special transcription factors. The WRKY domain contains a conserved WRKYGQK sequence followed by a Cys2His2 or Cys2HisCys zinc-binding motif (Eulgem et al., 2000). WRKY proteins recognize the W-box (T)TGAC(C/T) or W-like box *cis*-regulatory elements, which are often found in many defense gene promoters. In addition to the W-box, it can bind other *cis* elements, such as sugar-responsive element (AA/TAA) in barley and pathogen response element (TACTGCGCTTAGT) in rice (Cai et al., 2008, Cheng et al., 2015, Sun et al., 2003).

WRKY proteins have been reported to play broad and pivotal roles in plant-pathogen interactions and act in a complex signaling network as both positive and negative regulators of disease resistance (Chen and Chen, 2002, Eulgem et al., 2000, Li et al., 2004, Zheng et al., 2006). Till date, a total of 125 WRKY gene family members have been identified and uniquely named by the rice WRKY working group to avoid confusions in nomenclature (hereafter, we follow the nomenclature of rice WRKY working group) (Rice WRKY Working Group, 2012, Ross et al., 2007). Ryu et al. (2006) demonstrated that one-third of the 45 tested WRKY genes in rice were remarkably responsive to the inoculation of bacterial pathogen *Xanthomonas oryzae* pv. *oryzae* (*Xoo*) and the fungal pathogen *Magnaporthe grisea*, which indicated that WRKY genes are extensively involved in plant defense to pathogens (Ryu et al., 2006). Till date, at least 12 WRKY genes have been characterized to be involved in rice disease resistance through diverse mechanisms (Cheng et al., 2015). WRKY12 (originally named as WRKY03), WRKY13, WRKY22, WRKY30, WRKY55 (originally named as WRKY31), WRKY53, WRKY71, and WRKY104 (originally named as WRKY89) are positive regulators of rice resistance to pathogens. WRKY12, WRKY55, WRKY53, WRKY71, and WRKY104 could enhance rice resistance to *M*. *grisea* by up-regulating pathogenesis-related (PR) genes such as *NPR1*, *ZB8*, *POX22*.*3*, *PR1b*, *PBZ1,* and *Sci2* (Chujo et al., 2014, Liu et al., 2005, Liu et al., 2007, Zhang et al., 2008). Some of the WRKY-positive regulators were implicated in the biosynthesis and signaling of phytohormones such as salicylic acid (SA) and jasmonic acid (JA). For example, WRKY30 activated SA signaling genes in response to *M*. *grisea* infection, whereas WRKY13 activated a series of SA synthesis and signaling genes and simultaneously suppressed JA synthesis and responsive genes to enhance rice resistance to *M*. *grisea* and *Xoo* (Cheng et al., 2015, Qiu et al., 2007, Qiu et al., 2008). Reported negative regulators include WRKY28, WRKY42, WRKY62, and WRKY76. Most of them increase rice susceptibility to pathogens by suppressing the transcription of defense-related genes, phytoalexin synthesis-related genes, or resistance (*R*) genes (Cheng et al., 2015, Chujo et al., 2013, Peng et al., 2008, Yokotani et al., 2013). Notably, *WRKY45* positively regulates rice resistance to *M*. *grisea*, but it plays dual roles in rice resistance to bacteria via an alternative splicing model (Shimono et al., 2007, Shimono et al., 2012, Tao et al., 2009). Knockout of the variant *WRKY45-1* showed increased resistance to *Xoo* and *Xanthomonas oryzae* pv *oryzicola* (*Xoc*), which was accompanied by a higher level of SA and JA. On the contrary, the variant *WRKY45-2* was found to promote accumulation of JA, but not SA, and eventually play positive functions in response against *Xoo* and *Xoc*. The opposite roles of the two variants in rice-*Xoo* interaction are possibly attributed to their mediation of different defense signaling pathways.

Despite the fact that tremendous progresses on *WRKYs* have been achieved, it is believed that more *WRKY* members are involved in rice immunity, given the extensive involvement of *WRKYs* and complication of rice-pathogen interplays. The current study revealed that *Xoo*-inducible *WRKY72* negatively regulates rice resistance against bacterial blight. WRKY72 directly binds to the promoter of a JA biosynthesis enzyme gene *AOS1* and suppresses its transcription by recruiting DNA methylation on it. SAPK10-mediated phosphorylation on Thr129 of WRKY72 weakens its DNA-binding ability to *AOS1*, promotes the endogenous JA level, and finally enhances *Xoo* resistance.

## Results

### Transcription of *WRKY72* Is Induced by *Xoo* Infection and Exogenous JA

**Figure 1:**
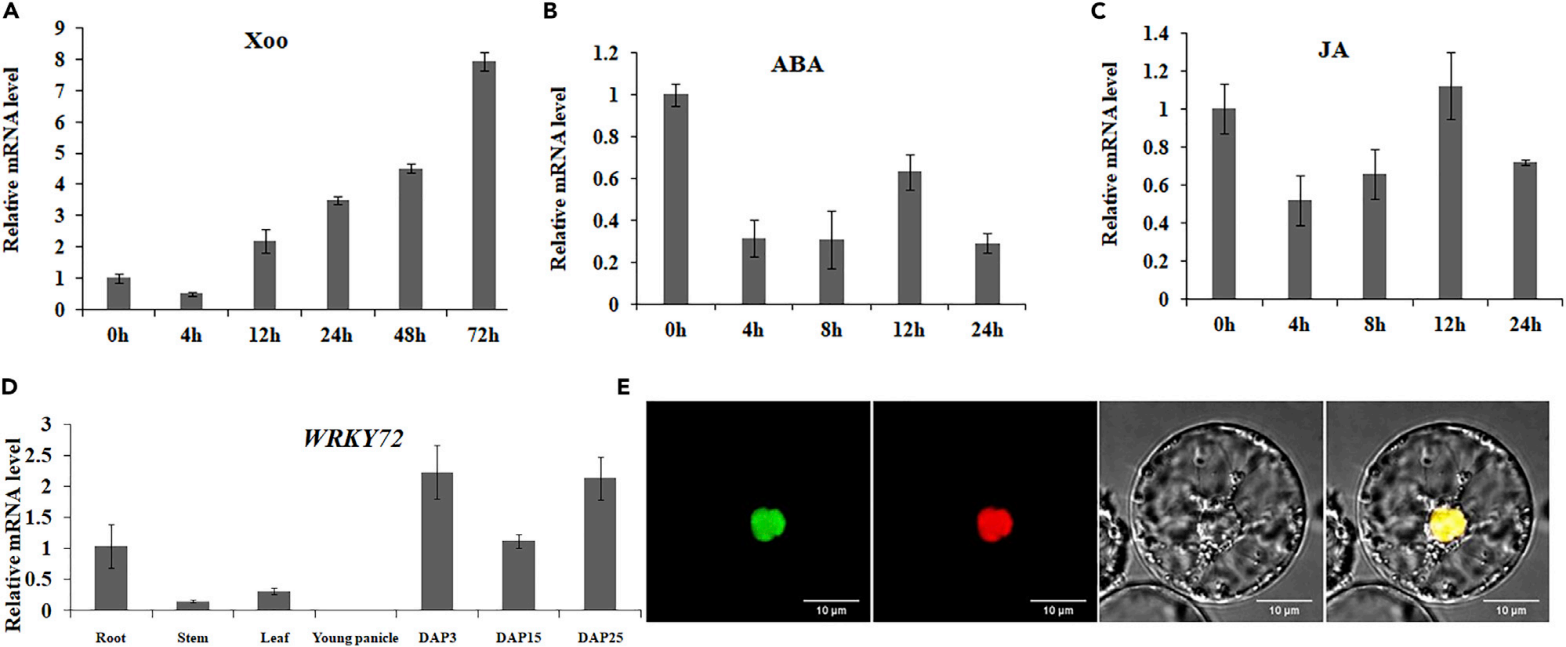
The Temporal-Spatial and Stress Expression Profiles of *WRKY72* (A–C) The time course expression level of *WRKY72* at *Xoo* inoculation and ABA and JA treatment. (A) *Xoo* and (B) ABA and (C) JA concentrations were ∼1 × 10^8^ colony-forming unit/mL and 100 μM, respectively. (D) The expression analysis of *WRKY72* in various tissues and stages. The expression level of leaves was set as 1. DAP: day after pollination. (E) Subcellular localization of WRKY72. 35S:WRKY72-GFP was co-transformed with a nuclear marker 35S:D53-mKate into protoplast. The fluorescent protein signals from left to right: 35S:WRKY72-GFP, 35S:D53-mKate, bright field, and merged. Scale bars, 10 μm. All data are shown as means ± SD of at least three biological replicates.

To find out the rice WRKYs involved in defense against bacterial blight, we performed qRT-PCR analysis of various *WRKYs* in a time line after *Xoo* inoculation and found that *WRKY72* (*LOC_Os11g29870*) is highly induced. The 3,736-bp-long gene encodes a 243-amino acid protein. In its only intron, we identified a SINE (short interspersed nuclear elements)-type transposon (2,812–3,024 bp) (http://www.repeatmasker.org/cgi-bin/WEBRepeatMasker). *WRKY72* displayed significantly elevated transcriptional level since 12 HAI (hours after inoculation) and reached highest level (over eight times up-regulation) at 72 HAI, when compared with the 0-HAI samples (Figure 1A). In addition, *WRKY72* also responds to treatment of exogenous phytohormones JA and ABA. *WRKY72* was immediately suppressed by ABA treatment at 4 and 8 HAI, and then increased to a higher level at 12 HAI (Figure 1B). JA treatment showed a similar induction pattern as ABA (Figure 1C). *WRKY72* is constitutively transcribed in various tissues, including leaf, root, panicle, callus, stem, and developing seeds (Figure 1D). To figure out the subcellular localization of WRKY72, we constructed a *pro35S*:WRKY72-GFP vector and co-transformed it with a marker nuclear protein *pro35S*:D53-mKate into rice protoplast. As expected, WRKY72 co-localized with D53 in the nucleus, which supported its functional annotation as a transcription factor (Figure 1E).

### *WRKY72* Suppresses Rice Resistance to *Xoo*

**Figure 2:**
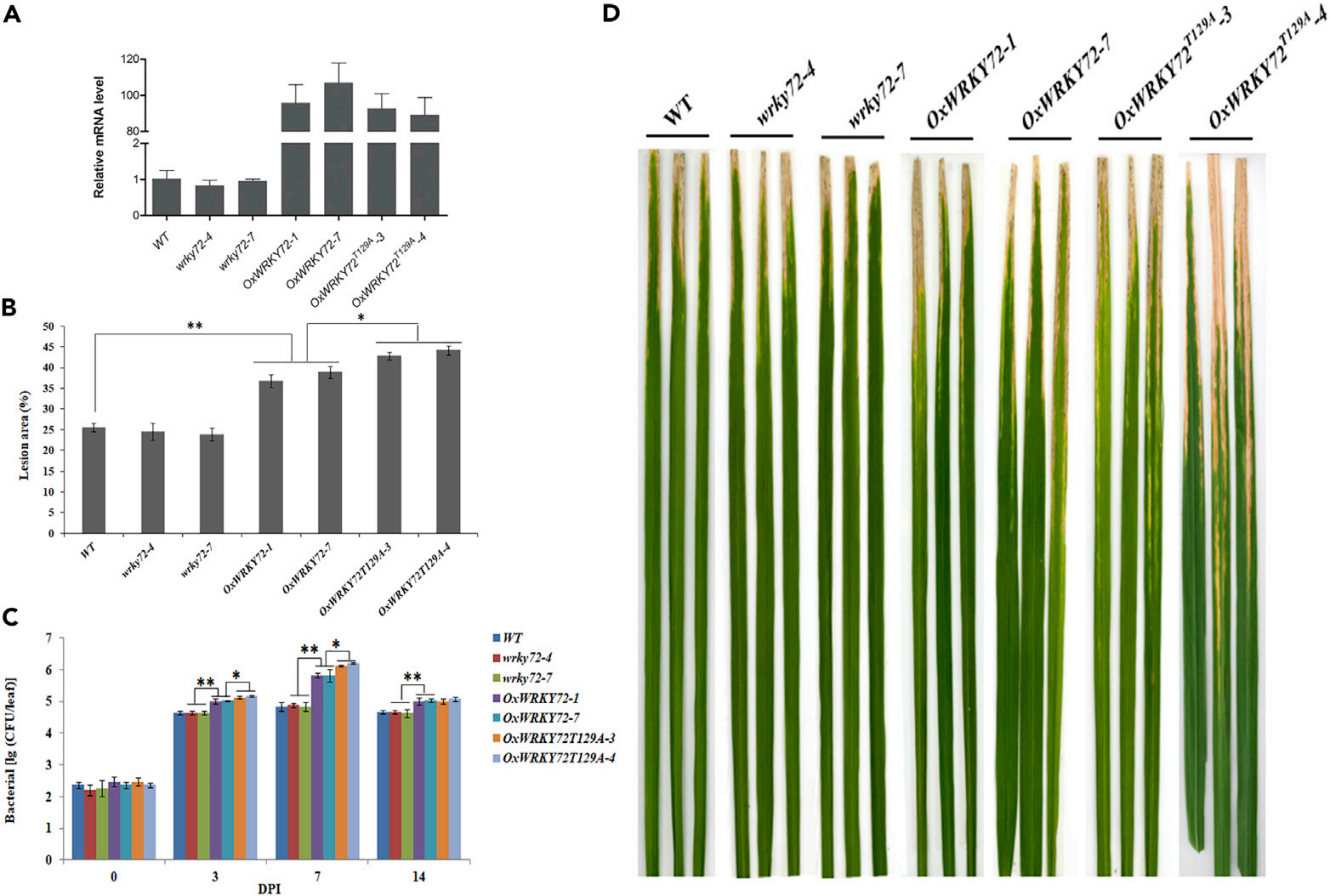
Phenotypical Characterization of *wrky72s*, *OxWRKY72s,* and WT against *Xoo* (A) The expression analysis of *WRKY72* in *wrky72*, *OxWRKY72,* and WT plant lines. (B–D) (B) The lesion area (%), (C) bacterial growth rate, and (D) necrosis lesion symposium in *wrky72*, *OxWRKY72,* and WT plant lines. *wrky72-4* and *wrky72-7*: *WRKY72* CRISPR/Cas9 lines; *OxWRKY72-1* and *OxWRKY72-7*: *WRKY72* over-expressing lines; *OxWRKY72*^*T129A*^*-3* and *OxWRKY72*^*T129A*^*-4*: *WRKY72* over-expressing lines with Thr129 substitution; WT: wild-type. Data are shown as means ± SD of at least three biological replicates. *p ≤ 0.05, **p ≤ 0.01 by the Student's t test.

To dissect the biological roles of *WRKY72* in rice bacterial blight resistance, we generated both CRISPR/Cas9-mediated mutant lines and over-expression lines of the gene. In T_1_ generation, two representative homozygous mutant lines *wrky72-4* and *wrky72-7* and two highly over-expressed lines *OxWRKY72-1* and *OxWRKY72-7* were used for phenotyping. *wrky72-4* and *wrky72-7* harbored a G and a T nucleotide insertion in the coding sequence, respectively, which should have disrupted the WRKY72 translation by shifting the open reading frame, although the transcription of the mutated genes were at the same level as native *WRKY72* (Figures 2A and S1). Both *OxWRKY72-1* and *OxWRKY72-7* showed over 80-fold increase of expression level. The major agronomic traits of the plants were largely the same, except that *OxWRKY72s* had lower yield per plant (Table S1). Upon artificial inoculation of *Xoo*, *OxWRKY72-1* and *OxWRKY72-7* became more susceptible than the wild-type (WT), as the lesion area on *OxWRKY72-1* and *OxWRKY72-7* were 37% and 39%, respectively, whereas 25% of the leaf area of WT displayed necrosis (Figures 2B and 2D). It was also revealed that the *Xoo* growth rates in *OxWRKY72s* were significantly higher than those in the WT at 3, 7, and 14 DPI (days post inoculation) (p < 0.05) (Figure 2C). All these disease index data demonstrated that *WRKY72* negatively regulated rice resistance to *Xoo*. However, *wrky72-4* and *wrky72-7* mutant lines displayed almost identical *Xoo* susceptibility as the WT did, which may be attributed to the functional redundancy of other sibling *WRKY* genes.

### WRKY72 Is Phosphorylated by SAPK10 at Thr129

**Figure 3:**
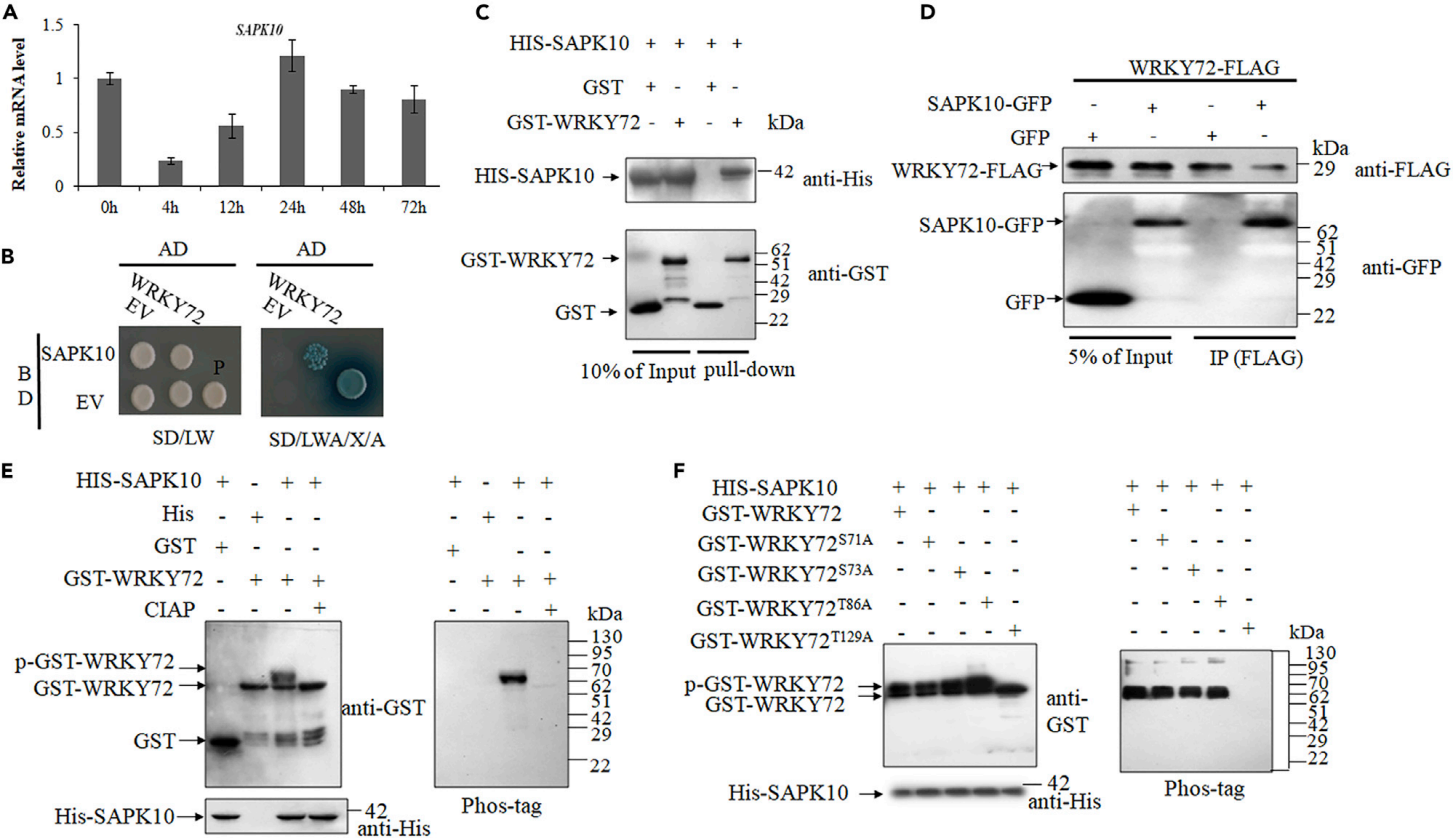
Protein-Protein Interaction and Phosphorylation Site Analysis of WRKY72 and SAPK10 (A) A time course expression level of *SAPK10* at *Xoo* inoculation. Data are shown as means ± SD of at least biological triplicates. (B) Yeast two-hybrid assays. BD: pGBKT7; AD: pGADT7; EV: empty vector; SD/LW: -Leu-Trp; SD/LWA/X/A: -Leu-Trp-Ade with the addition of X-α-Gal and aureobasidin A; P: positive control, pGADT7-T/pGBKT7-53. (C) *In vitro* pull-down of SAPK10 and WRKY72. His-SAPK10 and GST-WRKY72 and GST were expressed and purified in *E*. *coli* and subjected to GST pull-down assays, then detected by immunoblotting using anti-GST and anti-His antibodies, respectively. (D) *In vivo* co-immunoprecipitation (IP) assay of SAPK10 and WRKY72. GFP, SAPK10-GFP, and WRKY72-FLAG extracted from infiltrated *Nicotiana benthamiana* leaves were used in a coIP assay. Precipitates were immunoblotted with GFP and FLAG antibodies, respectively. (E and F) WRKY72 is phosphorylated by SAPK10 at Thr129. The recombinant protein GST-WRKY72 (E) and GST-WRKY72 with potential phosphosites substituted (F) were co-expressed with His-SAPK10 in *E. coli*, respectively. Equal amounts of the GST purified recombinant proteins were detected by immunoblotting using indicated antibodies. CIAP: Calf Intestine Alkaline Phosphatase; p-GST-WRKY72: phosphorylated GST-WRKY72; Phos-tag: biotinylated Phos-tag zinc BTL111 complex.

We screened over 1 million colonies from a cDNA library derived from rice young seedlings by using yeast two-hybrid method and detected an interactive kinase SAPK10 (LOC_Os03g41460). SAPK10 may be a negative regulator in response to *Xoo* infection, as its transcription was significantly suppressed by *Xoo* at the first 12 h after inoculation (Figure 3A). We repeated the yeast two-hybrid assay by a reciprocal hybridization and confirmed the SAPK10-WRKY72 interaction in yeast (Figure 3B). The interaction was further verified by pull-down *in vitro* and co-immunoprecipitation *in vivo* assays. The *in vitro* pull-down assay demonstrated that GST-WRKY72 protein could be pulled down by HIS-SAPK10 protein (Figure 3C). Meanwhile, SAPK10-GFP protein, but not the GFP tag control, was specifically co-immunoprecipitated with the WRKY72-FLAG in tobacco (Figure 3D). Thus, we proved the SAPK10-WRKY72 interaction in yeast, *in vitro* and *in vivo*. Subsequently, an *E*. *coli* kinase assay was performed to examine the kinase-substrate relationship between SAPK10 and WRKY72. When co-transformed with HIS-SAPK10, GST-WRKY72 showed a lagged band as detected by the GST antibody, indicating a phosphorylation has occurred on it (Figure 3E). The phosphorylation was further confirmed by the Phos-tag detection (Figure 3E). Therefore, we concluded that SAPK10 phosphorylates WRKY72 *in vitro*. To specify the SAPK10-dependent phosphosites on WRKY72, we generated the truncated forms of WRKY72 for the kinase assay. The phosphosite was gradually narrowed down to the fragment 1–135 with four potential sites (Figure S2), and finally localized at Thr129 (129^th^ threonine), which is out of, but very close to, the annotated WRKY domain (138–193 amino acid) (Figure 3F).

To test the effect of SAPK10-mediated phosphorylation on the function of WRKY72, we further generated *OxWRKY72*^*T129A*^ lines, in which the only phosphosite Thr129 was mutated into an alanine (Ala) to block the phosphorylation on it. Two *OxWRKY72*^*T129A*^ lines with comparable levels of *WRKY72* expression as the *OxWRKY72s* were chosen for *Xoo* susceptibility assay (Figure 2A). Interestingly, *OxWRKY72*^*T129A*^ lines exhibited higher susceptibility to *Xoo* than the *OxWRKY72* lines (Figure 2D), although the other major agronomic traits remained largely the same (Table S1), indicating that the phosphorylation on Thr129 might be a key switch to turn off the WRKY72 negative function in disease resistance.

### WRKY72 Regulates Defense-Related Genes and JA Biosynthesis Genes

**Figure 4:**
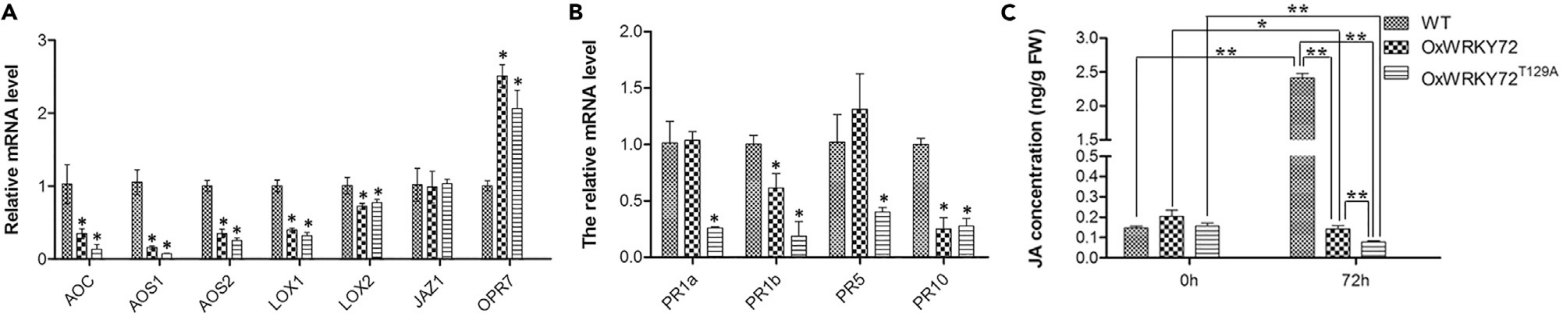
The Transcriptional Abundances of JA Biosynthesis and Pathogenesis-Related Genes and Endogenous JA Level Analysis in WT, *OxWRKY72,* and *OxWRKY72*^*T129A*^ (A and B) The transcriptional level of JA biosynthesis and pathogenesis-related genes in WT, *OxWRKY72,* and *OxWRKY72*^*T129A*^ at 72 h after *Xoo* infection. (C) The time course endogenous JA level in WT, *OxWRKY72,* and *OxWRKY72*^*T129A*^. WT: wild-type; *OxWRKY72*: *WRKY72* over-expression line 1 and line7 sample mixed; *OxWRKY72*^*T129A*^: *WRKY72* with Thr129 substitution form's over-expressing line 3 and 4 sample mixed. FW: fresh weight. Data are shown as means ± SD of three biological replicates. * And ** represent significant difference in the comparison at p ≤ 0.05 and p ≤ 0.01, respectively, as determined by the Student's t test.

To further elucidate the regulation network, we first performed RT-qCR to examine the transcriptional levels of a couple of JA synthesis genes in *OxWRKY72*, *OxWRKY72*^*T129A*^, and WT lines. The results suggested that the all the tested genes including *AOC* (*Allene Oxide Cyclase*, *LOC_Os03g32314*), *AOS1* (*Allene Oxide Synthase 1*, *LOC_Os03g55800*), *AOS2* (*Allene Oxide Synthase 2*, *LOC_Os03g12500*), *LOX1* (*Lipoxygenase 1*, *LOC_Os03g49380*), and *LOX2* (*Lipoxygenase 2*, *LOC_Os03g08220*) were significantly down-regulated in *OxWRKY72*, with the only exception of *OPR7* (*12-Oxophytodienoate reductase*, *LOC_Os08g35740*), which showed an opposite tendency (Figure 4A). More interestingly, the levels of *AOC*, *AOS1,* and *AOS2* were further reduced in *OxWRKY72*^*T129A*^ when compared with *OxWRKY72* (p < 0.05), which is in accordance with the observed higher susceptibility of *OxWRKY72*^*T129A*^. We also investigated the levels of PR protein genes *PR1a*, *PR1b*, *PR5,* and *PR10*. *PR1b,* and *PR10* may contribute to the elevated susceptibility of *OxWRKY72* and *OxWRKY72*^*T129A*^ lines, as both genes were significantly down-regulated (Figure 4B).

Given the obvious down-regulation of the key enzyme genes for JA biosynthesis, we examined the endogenous JA level in *OxWRKY72*, *OxWRKY72*^*T129A*^, and WT (Figure 4C). Before the *Xoo* infection, the three types of plants were in a similar and relatively low endogenous JA level, although *OxWRKY72* had a slightly higher level (p < 0.05). In the WT, *Xoo* infection significantly stimulated the accumulation of JA to about 16 times higher level than that in the pre-infection condition. However, the *Xoo* infection appeared to have triggered the suppression function of WRKY72 on JA synthesis, as we found that the JA level was significantly reduced in *OxWRKY72* at 72 HAI. Moreover, *OxWRKY72*^*T129A*^ displayed even lower JA level than *OxWRKY72* at 72 HAI, which is consistent with the observation that *OxWRKY72*^*T129A*^ lines were more susceptible to *Xoo* than *OxWRKY72s*. The results above suggested that WRKY72 negatively regulates *Xoo* resistance by suppressing JA biosynthesis.

### WRKY72 Directly Suppresses *AOS1* Transcription to Attenuate JA Biosynthesis

**Figure 5:**
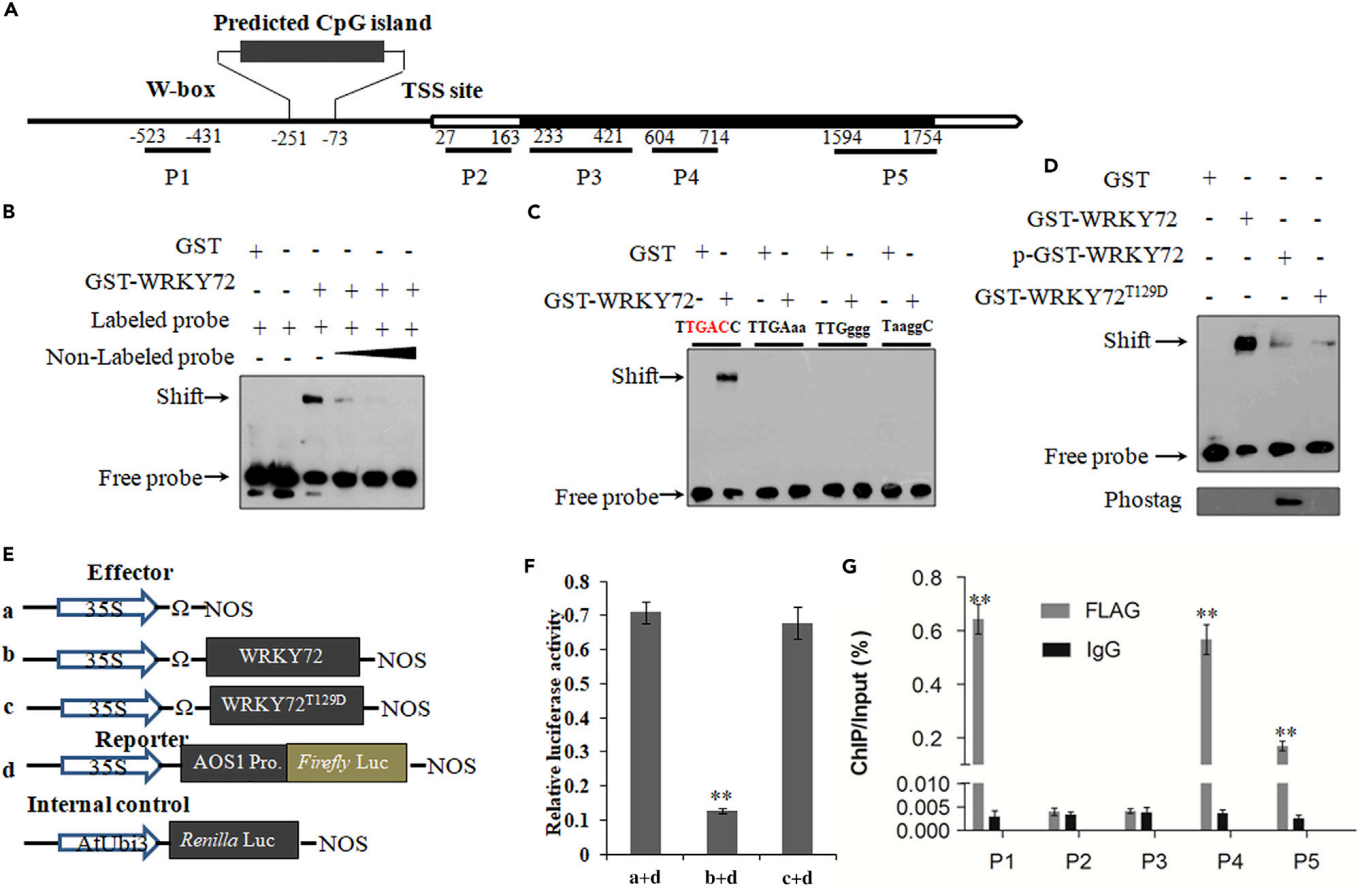
WRKY72 Directly Binds to the W-Box of *AOS1* Promoter and Suppresses Its Transcription, and Phosphorylation of WRKY72 can Reduce its Binding and Suppression Activity (A) Schematic presentation of the *AOS1* gene structures. Black boxes: exons; blank box: untranslated region; line: promoter. Transcription starting site (TSS) was set as 0. Numbers indicate the distances (bps) to the TSS. (B) EMSA assay showing WRKY72 could directly bind to the promoter of *AOS1*. The 5-, 10-, and 30-fold excess non-labeled probes were used for competition. (C) EMSA showing TTGACC is required by WRKY-72 binding to the promoter of *AOS1*. Probe sequence (60 bp) containing W-box (TTGACC). W-box region is shown in (A). TTGACC was substituted by TaaggC, TTGggg, and TTGAaa in the mutant probe, and the substitution nucleotide acids were marked with lowercases. (D) EMSA assay showing the binding between WRKY72 and *AOS1* is suppressed by phosphorylation of Thr129 in WRKY72 protein. GST-WRKY72, purified protein; p-GST-WRKY72: phosphorylated proteins; GST-WRKY72^T129D^: mimic phosphorylated proteins. The phosphorylated proteins were chemiluminescence detected by biotinylated Phos-tag zinc complex. (E) Scheme of the constructs used in LUC transient transcriptional activity assay. (F) LUC transient transcriptional activity assay in rice protoplast. Reporter: proAOS1:LUC; effectors: pro35S:WRKY72:tNOS and WRKY72^T129D^. The fLUC/rLUC ratio represents the relative activity of 35S promoter. (G) Validation of the direct binding of WRKY72 to the promoter and coding sequence (CDS) of *AOS1* by chromatin immunoprecipitation (ChIP)-qPCR. P1–P5 indicates the regions detected by ChIP-qPCR shown in (A).The enrichment values were normalized to Input. IgG-immunoprecipitated DNA was used as a control check (CK). All values are mean ± SD with biological triplicates. **p < 0.01 by the Student's t test.

The reduced level of endogenous JA and JA biosynthesis rate-limiting genes in *OxWRKY72* lines intrigued us to speculate that WRKY72 directly suppresses the transcription of these genes. To test this hypothesis, we checked the *in vitro* binding of WRKY72 on *AOC*, *AOS1,* and *LOX1* promoter regions by EMSA (electrophoretic mobility shift assay). The results showed that WRKY72 only bound to the promoter of *AOS1*, where a conserved W-box *cis* element exists (Figures 5A, 5B, and S3). The shifted bands were substantially weakened when non-labeled competitive probes were applied, suggesting a highly specific binding of WRKY72 to the promoter of *AOS1 in vitro* (Figure 5B). Meanwhile, the binding was completely impaired when the conserved W-box was mutated; hence this 6-nucleotide sequence (TTGACC) might be the core *cis* element for the binding of WRKY72 (Figure 5C). Moreover, we performed EMSA to investigate the DNA-binding ability of WRKY72 in different phosphorylation status. As shown in Figure 5D, p-GST-WRKY72 (Thr129 site phosphorylated) and WRKY72^T129D^, in which Thr129 was mutated to mimic constitutive phosphorylation status, both had substantially reduced signal of shifted bands when compared with the non-phosphorylated WRKY72. This observation suggested that SAPK10-mediated phosphorylation on Thr129 turns down WRKY72 function by weakening its DNA-binding ability. Subsequently, we generated *proUbi*:*WRKY72-FLAG* lines and used the leaves at 72 HAI for chromatin immunoprecipitation-qPCR assay. In total, five fragments representing the promoter, UTR, and coding sequence regions were examined, and we found that WRKY72 was significantly enriched in the W-box region of the *AOS1* promoter when compared with the mock, which proved the binding of WRKY72 to *AOS1* promoter *in vivo* (Figure 5G). Finally, we conducted a dual luciferase (LUC) transient transcriptional activity assay to test the effect of WRKY72 on *AOS1* transcription. When compared with the empty effector, *pro35S*:*WRKY72*:*tNOS* dramatically reduced the firefly LUC reporter level (Figures 5E and 5F), which is in agreement with the observed down-regulation of *AOS1* in *OxWRKY72* lines, indicating that WRKY72 directly suppresses *AOS1* transcription. In support to the observed lower DNA-binding ability of WRKY72^T129D^ in EMSA, we also found that the suppression on *AOS1* was significantly reduced when WRKY72^T129D^ was used as the effector (Figures 5E and 5F).

**Figure 6:**
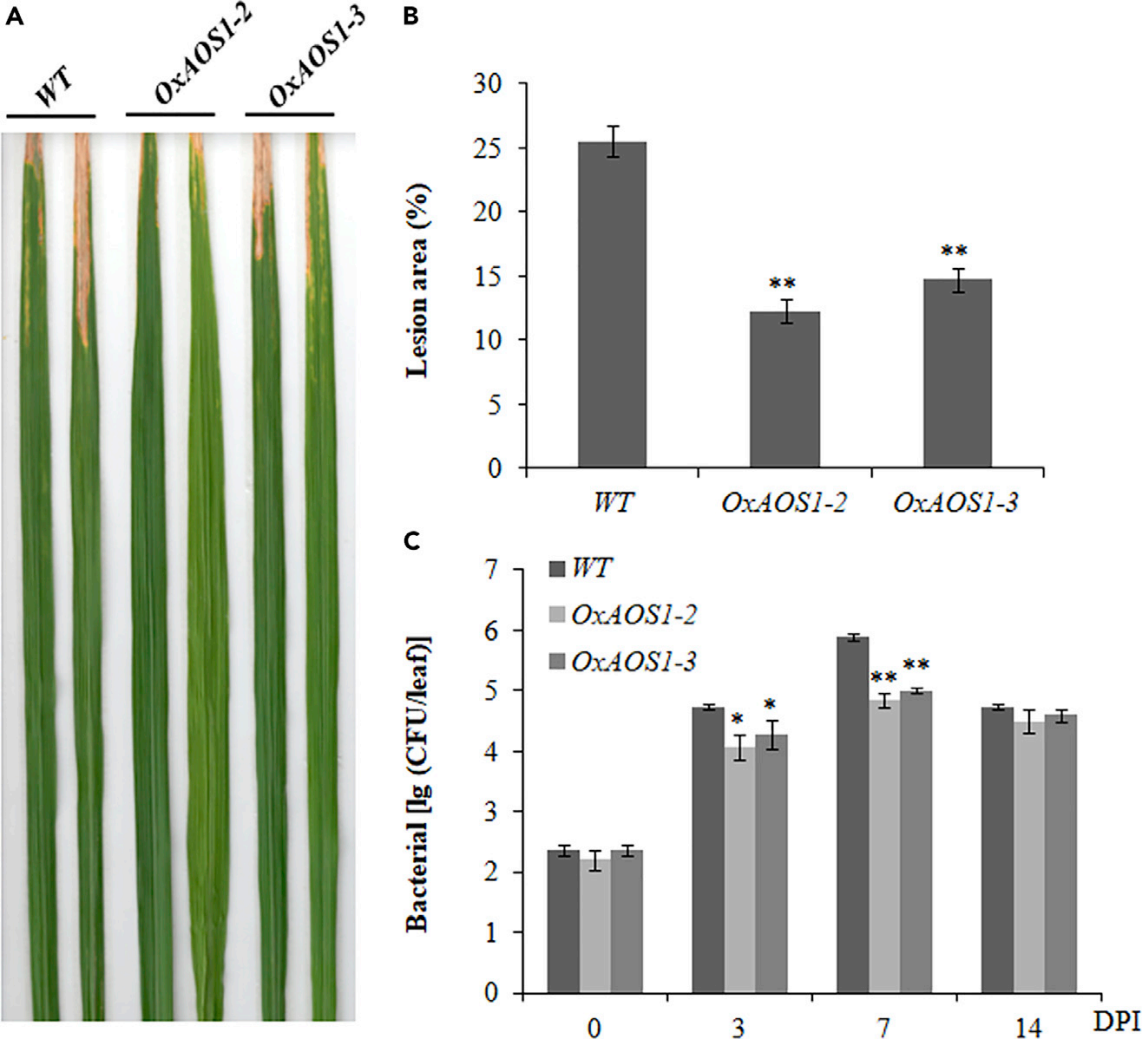
Phenotypical Characterization of *OxAOS1s* and WT against *Xoo* (A–C) Necrosis lesion symposium (A), the lesion area (%) (B), and bacterial growth rate (C) in *OxAOS1* and WT plant lines. *OxAOS1-2* and *OxAOS1-3*: *AOS1* over-expressing lines; WT: wild-type. Data are shown as means ± SD of at least three biological replicates. *p ≤ 0.05, **p ≤ 0.01 by the Student's t test.

To figure out the biological functions of *AOS1* in rice disease resistance, we further over-expressed *AOS1* in Nipponbare background. The *OxAOS1* lines had substantially elevated transcription level of *AOS1* and became dwarf, which is a typical phenotype of plants with high endogenous JA levels, given that JA generally represses plant growth (Figures S4A–S4C). After the artificial inoculation of *Xoo*, *OxAOS1* lines exhibited less lesion areas in the leaf and lower bacterial growth rate, suggesting a positive role of *AOS1* in response to *Xoo* infection (Figures 6A–6C).

### WRKY72 Induces DNA Hypermethylation on *AOS1* Promoter

**Figure 7:**
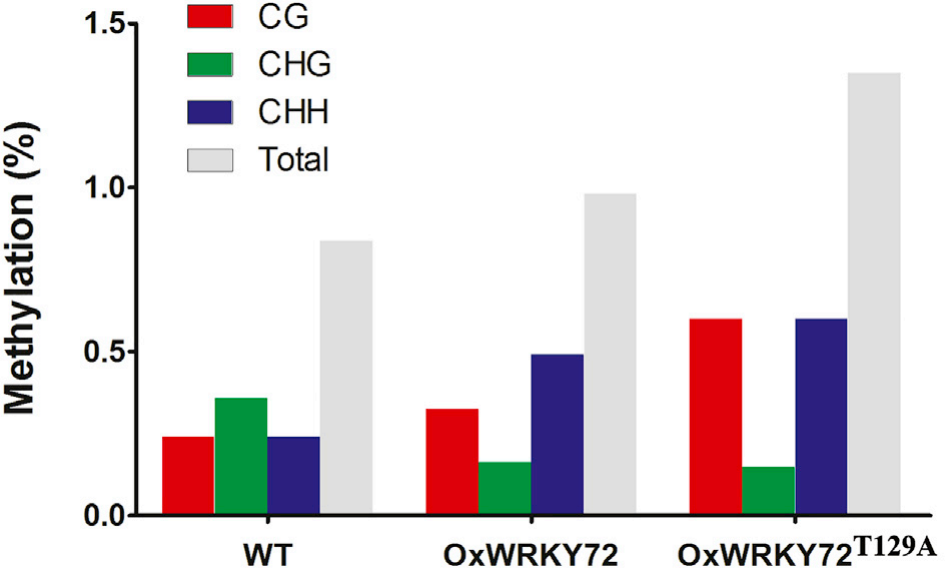
DNA Methylation Patterns and Level in WT, *OxWRKY72,* and *OxWRKY72*^*T129A*^ CG, CHG and CHH: three methylation patterns; WT: wild-type; *OxWRKY72*: *WRKY72* over-expression line 1 and line7 sample mixed; *OxWRKY72*^*T129A*^: *WRKY72* with Thr129 substitution form's over-expressing line 3 and 4 sample mixed. DNA methylation region detected is shown in Figure 5A.

DNA methylation has been revealed as a profound epigenetic mechanism involved in gene repression. To address the question that how is *AOS1* suppressed by WRKY72, we checked the DNA methylation pattern and level on the *AOS1* promoter region (minus 251 – minus 73), which is the only potential CpG island as predicted by MethPrimer (http://www.urogene.org/cgi-bin/methprimer/methprimer.cgi) (Figure 5A). When compared with the WT, the CG and CHH levels of *OxWRKY72* were significantly increased, which led to a significant increase of the total DNA methylation level, although the CHG level was decreased (Figure 7). *WRKY72*^*T129A*^ exhibited hypermethylation on this region, when compared with WT and *OxWRKY72* (Figure 7). The correlation between the DNA methylation and *AOS1* transcription levels suggested that WRKY72 may induce DNA hypermethylation on *AOS1* promoter to suppress its transcription.

**Figure 8:**
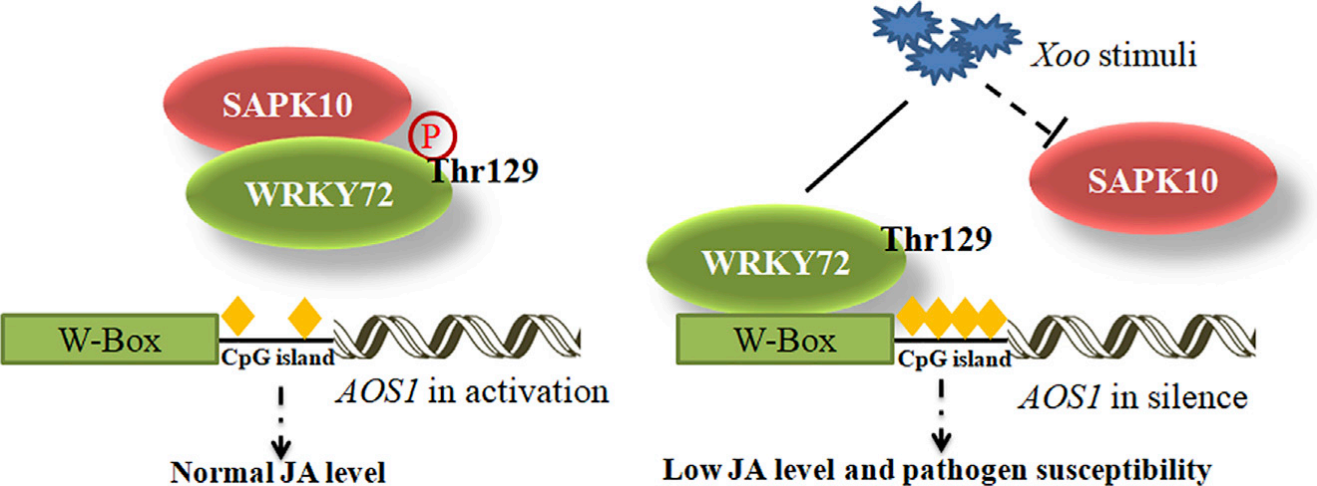
Working Model of WRKY72-Mediated Regulation against *Xoo* Infection in Rice Line and dotted line indicate positive and negative regulation in transcription, respectively; ⓟ indicates the phosphorylation status of the protein; and yellow rhombus indicates DNA methylation on the region.

From the results above, a working model of WRKY72 was proposed (Figure 8). Under normal growth conditions, WRKY72 is phosphorylated by SAPK10 at Thr 129, which releases the suppression of WRKY72 to *AOS1* by impairing WRKY72 DNA-binding ability and lowering the DNA methylation level on *AOS1* promoter, thus maintaining a normal endogenous JA level for growth. Under *Xoo* infection, the stimuli represses *SAPK10* transcription and makes the WRKY72 in a non-phosphorylated status, which facilitates the binding of WRKY72 to the W-box *cis* element of *AOS1* promoter to suppress *AOS1* transcription by recruiting hyper DNA methylation on it, and eventually contributes to plant susceptibility by suppressing the endogenous JA biosynthesis (Figure 8). It should be noted that, although our working model proposed a suppressing pathway of JA biosynthesis induced by *Xoo* infection, we did observe that the final endogenous JA level in Nipponbare plants was drastically increased by *Xoo* stimuli at 72 HAI (Figure 4C). Such a phenomenon suggested the existence of a feedback loop comprising suppressing pathways such as SAPK10-WRKY72-AOS1 as well as some unknown activating pathways of JA biosynthesis, whose counterbalance finally decides the endogenous level in rice. In the time point for JA quantification assay, possibly due to the weak level of WRKY72 in WT plants, the WRKY72-mediated suppression might be overcounted by the JA biosynthesis activation pathways, which eventually gave rise to the elevated JA level in the WT plant. Nevertheless, in the OxWRKY72 lines with magnified WRKY72 effects, the suppression pathway overrode the activating pathways and finally led to lower JA level and pathogen susceptibility after *Xoo* infection, which perfectly matched the JA quantification assay result in Figure 4C.

## Discussion

### WRKY72 Suppresses Endogenous JA Level in Defense

It has been well known that JA, as an activating signal molecule, triggers immunity to confer broad-spectrum resistance for plants (Okada et al., 2015). Pathogen infection or other forms of biotic attack stimulate rapid biosynthesis of JA and its derivatives, which would promote the expression of defense-related proteins and secondary metabolites such as alkaloids, terpenoids, and PR proteins (Campos et al., 2014). Meanwhile, genetically knocking out or knocking down JA biosynthesis or signaling genes led to higher susceptibility of the plants to various pathogens. In *Arabidopsis*, disruption of JA receptor gene *Coi1* or JA-lle synthesis gene *JAR1* makes the plants susceptible to necrotrophic pathogens or soil fungus, respectively (Staswick et al., 2002, Staswick et al., 2010). Likewise, rice jasmonate-deficient plants *cpm2* and *hebiba* were found to lose their resistance to an originally incompatible avirulent strain of *M*. *grease*, whereas ectopic expression of *AOS2* encoding a JA production enzyme enhanced the plant resistance to pathogenic fungi (Mei et al., 2006, Riemann et al., 2013). JA may also promote plant resistance to hemi-necrotrophic *Xoo*. For example, JA signaling genes *JAZ8* and *MYC2* both are involved in rice resistance to bacterial blight (Uji et al., 2016, Yamada et al., 2012).

WRKYs are very important transcription factors in plants. The majority of over 100 members in rice are found to be involved in plant defense response in either a negative or a positive manner. The negative regulator members include WRKY28, WRKY42, WRKY62, and WRKY76, but their regulatory mechanism may vary from each other. WRKY62 and WRKY76 cause pathogen susceptibility by regulating a list of defense-related genes or interacting with the intracellular kinase domain of Xa21 to affect its protein cleavage and nuclear localization (Park and Ronald, 2012, Peng et al., 2008, Yokotani et al., 2013). Recently, emerging evidences linked the WRKY-regulated pathogen response to JA accumulation or signaling. For example, WRKY42-knockdown and WRKY42-over-expressing plants showed increased resistance and susceptibility to *M*. *oryzae*, which are accompanied by increased and reduced JA content, respectively (Cheng et al., 2015). In this research, we found that WRKY72 negatively regulates rice response to *Xoo* infection by suppressing JA biosynthesis, as the WRKY72 over-expression lines became more susceptible upon *Xoo* inoculation. A couple of JA biosynthesis genes were significantly down-regulated in *OxWRKY72* lines. Moreover, we provided several layers of *in vivo* and *in vitro* evidences to show that WRKY72 directly binds to the conserved W-box *cis* element of JA biosynthesis rate-limiting enzyme gene *AOS1* and suppresses *AOS1* transcription, which eventually reduced endogenous JA level and rice resistance to bacterial blight. AOS enzymes catalyze the conversion of 13-HPOT (13-hydroperoxy-9,11,15-octadecatrienoic acid) to 12,13-EOT ((9Z,11E,15Z,13S,12R)-12,13-epoxy-9,11,15-octadecatrienoic), which is the first step toward JA biosynthesis (Schaller, 2001). Among the four *AOS* genes in rice (*AOS1*-*AOS4*), *AOS2* has been characterized as a pathogen-inducible gene, and its over-expression lines had higher levels of JA and stronger resistance to *M*. *grisea* (Mei et al., 2006). Likewise, the current study revealed that over-expression of *AOS1* also enhanced plant resistance to *Xoo* infection, when compared with the WT. In another study, *AOS1* was isolated by positional cloning as *Pre* (*precocious*) controlling juvenile-to-adult phase transition in rice. *Pre* exhibited long leaf with precociously acquired adult features in midrib formation, shoot meristem size and plastochron, and more importantly, lower endogenous JA level (Hibara et al., 2016). Although the authors did not explore the potential roles of *AOS1* in disease resistance, it is rational to expect a higher susceptibility of *aos1* to *Xoo* infection.

### SAPK10-Mediated Phosphorylation Turns Down WRKY72 Function as a Repressor

Post-translational modifications, particularly protein phosphorylation, have been long recognized as a significant regulatory mechanism controlling transcription factor activity (Meng et al., 2013, Yang et al., 2017). In plant defense, MAPK (mitogen-activated protein kinase) is a major type of kinase that can phosphorylate disease resistance-related transcription factors such as WRKYs. Phosphorylation within the SP cluster of WRKY proteins by MAPKs is thought to exert a booster function in the expression of downstream genes (Asai et al., 2002, Ishihama and Yoshioka, 2012, Pitzschke et al., 2009). In *Arabidopsis*, AtMPK3 and AtMPK6 directly phosphorylated AtWRKY33 to enhance the production of phytoalexin camalexin and phytohormone ethylene, whereas non-phosphorylated AtWRKY33 was not able to fully rescue *wrky33* mutant, implying that MAPK-dependent phosphorylation activates AtWRKY33 function (Li et al., 2012, Mao et al., 2011, Wang et al., 2018). Similarly, Chujo et al. (2014) found that WRKY53-mediated resistance to rice blast fungus strain Ina86-137 relied on the phosphorylation on its serine-proline residues by MPK3/MPK6. Over-expressing a phosphomimic mutated version of WRKY53 (WRKY53SD) rice plants elevated the expression level of defense-related genes and enhanced disease resistance to *M*. *oryzae* compared with native WRKY53-over-expressing rice plants (Chujo et al., 2014). It was suggested that the positive effect of MAPK-dependent phosphorylation on WRKYs might be achieved by increasing its DNA-binding activities on target genes (Ishihama and Yoshioka, 2012, Koo et al., 2009, Menke et al., 2005).

In this study, we demonstrated that SAPK10 kinase cloud physically binds to and phosphorylates WRKY72 at Thr129. In contrast to the above-mentioned cases that phosphorylation activated WRKYs, the phosphorylation on WRKY72 weakened its DNA-binding ability to *AOS1* promoter, to thus release the inhibition on JA accumulation. In support of this finding, the *OxWRKY72*^*T129A*^ lines, which had blocked SAPK10 target phosphosite, showed drastically reduced transcription of downstream JA biosynthesis genes, endogenous JA level, and resistance to *Xoo* infection than the native *OxWRKY72* lines. Hence, the indication is that the SAPK10-dependent phosphorylation on WRKY72 turns down its function as a transcription repressor in plant defense, representing a diverse mechanism to the previously reported MAPK-WRKY module. SAPK10 is an ABA-inducible SnRK2-type kinase involved in ABA signaling (Kobayashi et al., 2010). So far, the effects of ABA on plant disease resistance remain elusive. ABA likely plays negative roles in plant defense; however, the interplay of ABA with other phytohormones often produces complicated network and possibly promotes defense in plants (Ton et al., 2009). Notably, a couple of rice SnRK2s have been implicated in response to *Xoo* infection (Xu et al., 2013). Our results hint a pathway “SAPK10-WRKY72-AOS1” in the cross talk of ABA-JA as well as in the ABA-mediated plant defense response, which will be further explored in our future study.

### WRKY72 Recruits DNA Hypermethylation to Repress *AOS1*

DNA cytosine methylation is usually connected with transcriptional silence of the target genes in numerous biological processes, including plant defense (Chen and Zhou, 2013, Wang et al., 2018). RdDM (RNA-directed DNA methylation) is a major mechanism to recruit DNA methyltransferases to the target site to execute DNA methylation. In such a case, DNA methylation is guided by a series of 21- to 24-nt small interfering RNA (siRNAs) with high homology with the target sites. The siRNAs could be derived from viral replication intermediates, products of endogenous RNA-directed RNA polymerase, transcribed inverted repeats, or TEs (transposable elements) (Wassenegger et al., 1994). One of the well-documented RdDM cases in rice defense is TE-siR815 (Zhang et al., 2016). TE-siR815 is an siRNA that originates from a MITE (miniature inverted repeat transposable elements). TE existed in the first intron of *WRKY45*, whose two variants *WRKY45-1* and *WRKY45-2* play opposite roles in response to *Xoo* and *Xoc* infection (Tao et al., 2009). Only the negative player *WRKY45-1* produces TE-siR815, which imposes DNA hypermethylation on *ST1* via an RdDM mechanism to abolish WRKY45-mediated pathogen resistance (Zhang et al., 2016). In our study, the total DNA cytosine methylation level on *AOS1* promoter was negatively correlated with the *AOS1* transcription level and endogenous JA level in WT, *OxWRKY72,* and *OxWRKY72*^*T129A*^ lines, implying that the repressor role of WRKY72 may be achieved by inducing DNA methylations on the promoter region of its direct target *AOS1*. Nevertheless, the question that how was the DNA methyltransferase recruited to the target sites remain to be addressed. The identified SINE TE in *WRKY72* intron might be a good clue that WRKY72 suppresses target genes through an RdDM mechanism, given the reported example of its homolog WRKY45 and many SINE-directed DNA methylation cases in mouse and human beings (Estécio et al., 2012, Yates et al., 2003).

### Limitations of the Study

In this study, we revealed a “SAPK10-WRKY72-AOS1” module, through which *Xoo* infection suppresses JA biosynthesis to cause *Xoo* susceptibility. However, as we have discussed in the article, the final endogenous JA level in plant is determined by the counterbalance of both activation and suppression pathways. Therefore, figuring out the *Xoo*-activated JA biosynthesis regulatory pathways would be of great interest for us to elucidate the comprehensive reaction of rice in response to *Xoo* infection. In addition, although we provided clues that WRKY72 represses *AOS1* transcription via RdDM, the detailed mechanism needs to be explored in future studies.

## Methods

All methods can be found in the accompanying Transparent Methods supplemental file.

## Supplemental Information

Document S1. Transparent Methods, Figures S1–S4 and Tables S1 and S2
