# Supplementary material for: SAPK10-Mediated Phosphorylation on WRKY72 Releases Its Suppression on Jasmonic Acid Biosynthesis and Bacterial Blight Resistance
Source: iScience. 2019 Jun 11;16:499–510. doi: 10.1016/j.isci.2019.06.009 (PMC6593165; doi:10.1016/j.isci.2019.06.009)
Supplement: Document S1. Transparent Methods, Figures S1–S4 and Tables S1 and S2 [file mmc1.pdf]

ISCI, Volume *16*

Supplemental Information

SAPK10-Mediated Phosphorylation on WRKY72 Releases Its Suppression on Jasmonic Acid Biosynthesis and Bacterial Blight Resistance

Yuxuan Hou, Yifeng Wang, Liqun Tang, Xiaohong Tong, Ling Wang, Lianmeng Liu, Shiwen Huang, and Jian Zhang

Supporting information

Figure S1. Sanger Sequencing of the Mutated Sites in Homozygous Mutants of *wrky72-4 and wrky72-7*, Related to Figure 2.

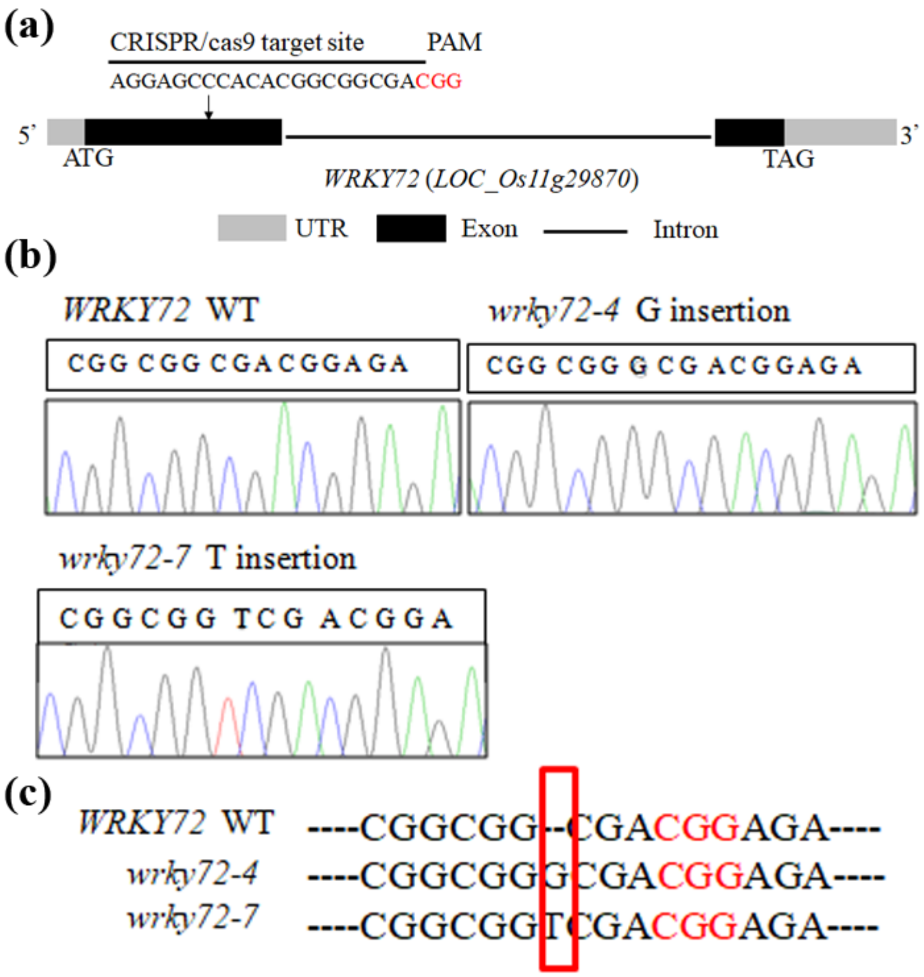

(a) Schematic presentation of the *WRKY72* structure and gene editing site. (b) Sanger sequencing chromatograph of the target site on *WRKY72*. (c) Summary of the mutations in the gene edited lines.

Figure S2. *In vitro* Phosphorylation of the Gradually Narrowed Fragment of WRKY72, Related to Figure 3.

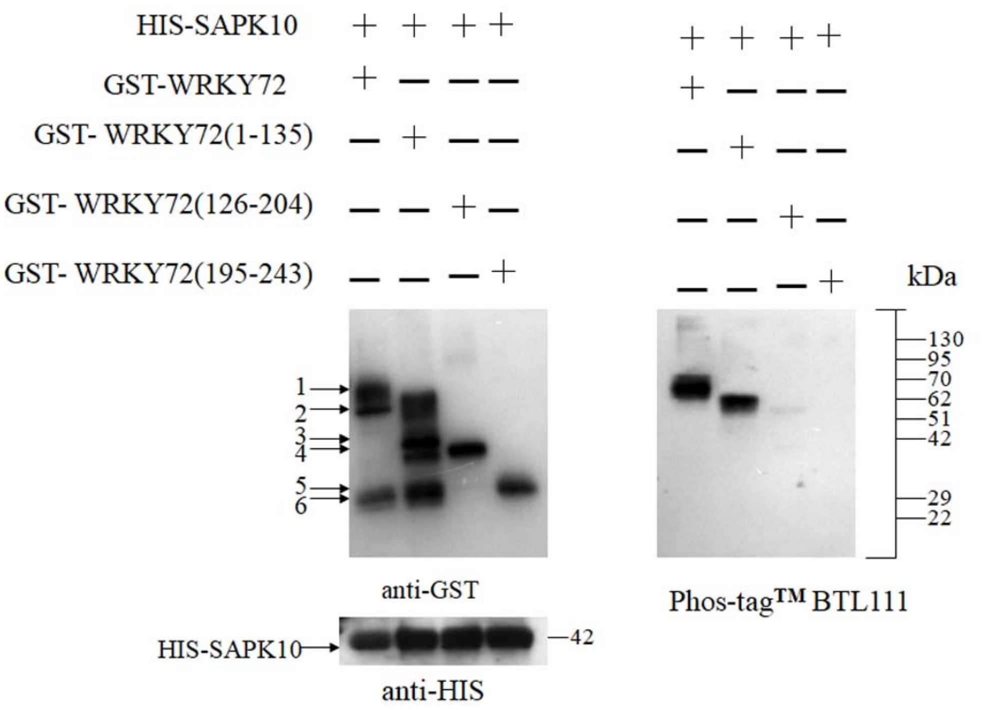

1:p- WRKY72; 2:GST-WRKY72;3. GST- WRKY72(1-135);4:GST- WRKY72(126-204);5:GST-WRKY72(195-243); 6.GST.

Figure S3. EMSA of WRKY72 on *AOC* and *LOX1* Promoter Regions, Related to Figure 5.

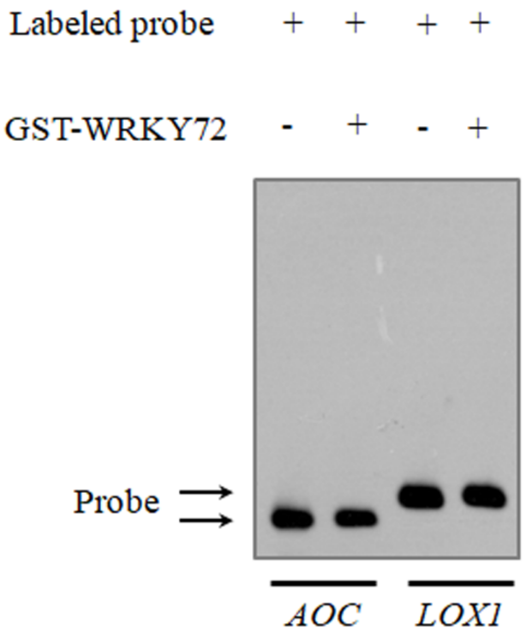

Figure S4. Major Agronomic Traits of *OxAOS1s* and WT, Related to Figure 6.

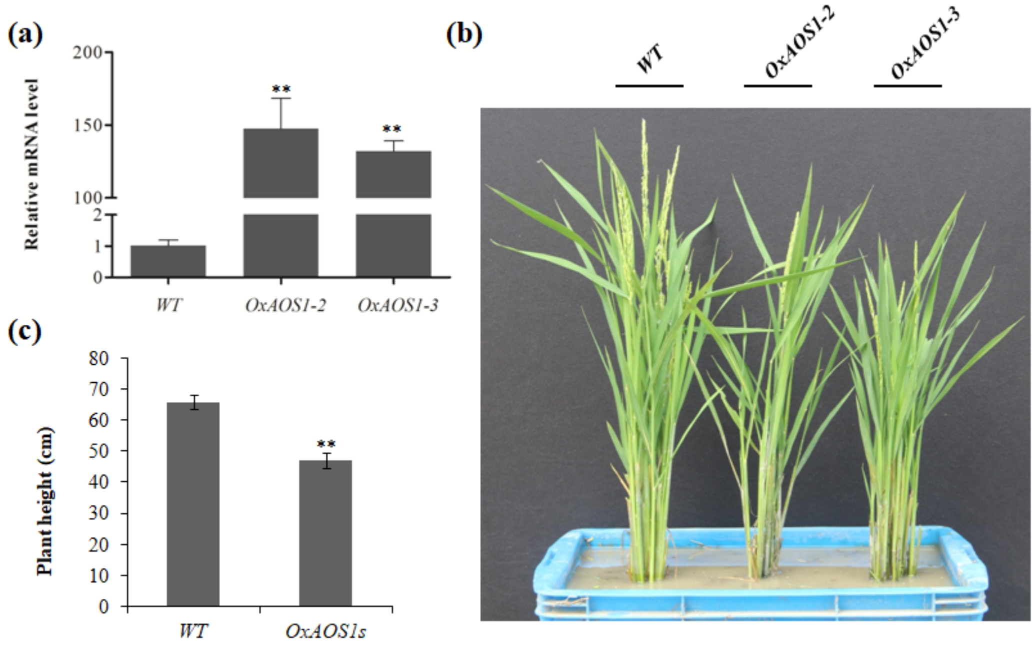

(a) The expression analysis of *AOS1* in *OxAOS1* and WT plant lines. (b-c) The height analysis of *OxAOS1* and WT plant lines. Data are shown as means ± SD of at least three biological replicates. *: P≤0.05, **≤0.01 by the Student's *t* test.

Table S1. Major Agronomic Traits of *WRKY72* CRISPR and Over-expressing Plants, Related to Figure 2.

	Plant height (cm)	Flowering date (days)	Seed length (cm)	Seed width (cm)	Yield /plant (g)
WT	69.7±1.06	72.0±2.00	7.7±0.10	3.4±0.10	30.57±0.53
wrky72-4	69.3±2.17	70.0±1.00	7.8±0.31	3.4±0.21	29.97±1.49
wrky72-7	69.9±1.07	70.7±0.58	7.6±0.15	3.3±0.15	31.82±1.39
OxWRKY72-1	68.7±1.20	70.3±1.53	7.4±0.40	3.5±0.06	27.93±0.46*
OxWRKY72-7	68.6±1.15	71.3±1.53	7.6±0.25	3.4±0.15	26.63±1.27*
$OxWRKY72^{T129A}$-3	69.9±2.18	72.0±1.73	7.7±0.21	3.4±0.10	26.58±0.72**
$OxWRKY72^{T129A}$-4	68.6±1.18	71.7±2.08	7.5±0.32	3.3±0.23	26.22±0.95**

The data is presented as the means ± SD of at least three biological replicates. The significant difference between the WT and *WRKY72* CRISPR and over-expressing plants is determined by the Student's *t* test, the single asterisk indicates P≤0.05, and double asterisks indicate P≤0.01. WT: wild-type; *wrky72-4* and *wrky72-7*: *WRKY72* CRISPR plants; *OxWRKY72-1* and *OxWRKY72-7*: *WRKY72* over-expressing plants; $OxWRKY72^{T129A}$-3 and $OxWRKY72^{T129A}$-4: *WRKY72* over-expressing plants with Thr129 substituted by Alanine.

Table S2. Sequences of Primers Used in This Study, Related to Figure 1, 2, 3, 4, 5, 6 and 7.

Name	Gene ID	Primer sequence (5' to 3')
**Primers for over-expression constructs**		
WRKY72-KpnI-F	LOC_Os11g29870	GGGGTACCATGGAGAACTTCCCGATACTCTTTG
WRKY72-BamHI-R		CGGGATCCCTACTGGAACATGTGGGAAGCAGCA
WRKY72-FLAG-KpnI-F		AAAGGTACCATGGAGAACTTCCCGATACTCTTTG
WRKY72-FLAG-BamHI-R		CGGGATCCCTAGGGCCCCCCCTCGACTTTATCGTCA
OxAOS1F	LOC_Os03g55800	TACGAACGATAGCCGATGGCCACGGCGGCGGCT
OxAOS1R		GGACTCTAGAGGATCTCAGAAGGTGGCCTTCTTG
**Primers for subcellular localization construct**		
ScWRKY72F		CAGTGGTCTCACAACATGGAGAACTTCCCGATACT
ScWRKY72R		CAGTGGTCTCATACACTGGAACATGTGGGAAGCAG
**Primers for qRT-PCR**		
qWRKY72F	LOC_Os11g29870	CAAGGGTGCAACGTGAAGAA
qWRKY72R		ATTTCTCGATGGGGTGCGTG
qAOCF	LOC_Os03g32314	GCCAAGGTGCAGGAGATGTTCG
qAOCR		AGCCGCTTGTCCAGGCTTCC
qAOS1F	LOC_Os03g55800	CGGGACATGGTGGTGGAGA
qAOS1R		GGAGTCGTATCGGAGGAAGAGC
qAOS2F	LOC_Os03g12500	TCGTCGGAAGGCTGTTGC
qAOS2R		ACGATTGACGGCGGAGGT
qLOX1F	LOC_Os03g49380	CCAACCAGACAAAGGCAGTA
qLOX1R		GGGAGAACACCCTCAACAATA
qLOX2F	LOC_Os08g39840	CGACGACCGTGTCTACGACTA
qLOX2R		CGTCTCCGACTTAGGGTCTTTT
qOPR7F	LOC_Os08g35740	GAAGGTGGTGGATGCTGTT
qOPR7R		TTTAGGATACTTGCCATAGGAG
qPR1aF	LOC_Os07g03710	CGTGTCGGCGTGGGTGT
qPR1aR		GGCGAGTAGTTGCAGGTGATG
qPR1bF	LOC_Os01g28450	TACGCCAGCCAGAGGAGC
qPR1bR		GCCGAACCCCAGAAGAGG
qPR5F	LOC_Os12g43430	TACAACGTCGCCATGAGCTTC
qPR5R		ACTTGGTAGTTGCTGTTGCCG
qPR10F	LOC_Os12g36830	CCATGAAGCTTAACCCCGATG
qPR10R		AGCTTGCCCACCTTGCTTT
**Primers for yeast two-hybrid**		
SAPK10-EcoRI-F	LOC_Os03g41460	CGGAATTCATGGACCGGGCGGCGCTGACGGTGG

SAPK10-PstI-R		AACTGCAGTCACATAGCGTATACTATCTCCCCA
WRKY72-EcoRI-F		CGGAATTCATGGAGAACTTCCCGATACTCTTTG
WRKY72-BamHI-R		AAAGGATCCCTACTGGAACATGTGGGAAGCAGCA
**Primers for pull-down**		
WRKY72-BamHI-F		AAAGGATCCATGGAGAACTTCCCGATACTCTTTG
WRKY72-SmaI-R		AAACCCGGGAACTACTGGAACATGTGGGAAGCAGCA
SAPK10-EcoRI-F		CGGAATTCATGGACCGGGCGGCGCTGACGGTGG
SAPK10-XhoI-R		CCGCTCGAGTCACATAGCGTATACTATCTCCCCA
**Primers for co-IP**		
WRKY72-XbaI-F		GCTCTAGAATGGAGAACTTCCCGATACTCTTTG
WRKY72-SmaI-R		TCCCCCGGGCTGGAACATGTGGGAAGCAGCAGCA
SAPK10-KpnI-F		GGGGTACCATGGACCGGGCGGCGCTGACGGTGG
SAPK10-XbaI-R		GCTCTAGACATAGCGTATACTATCTCCCCA
**Primers for WRKY72 mutation (overlap-PCR)**		
WRKY72-S71A-mutation-F		GGAGGAGCTCGCCAACTCCAAGCAG
WRKY72-S71A-mutation-R		GCTTGGAGTTGGCGAGCTCCTCCAT
WRKY72-S73A-mutation-F		GCTCTCCAACGCCAAGCAGGCCGGC
WRKY72-S73A-mutation-R		CGGCCTGCTTGGCGTTGGAGAGCTC
WRKY72-T86A-mutation-F		CGGTGGTGCAGCCAGGAGCCCACAC
WRKY72-T86A-mutation-R		GTGGGCTCCTGGCTGCACCACCGTC
WRKY72-T129A-mutation-F		CGCGTTCCAGGCCCGCAGCCAGGTC
WRKY72-T129A-mutation-R		CCTGGCTGCGGGCCTGGAACGCGAA
WRKY72-T129D-mutation-F		CGCGTTCCAGGACCGCAGCCAGGTC
WRKY72-T129D-mutation-R		CCTGGCTGCGGTCCTGGAACGCGAA
**Primers for EMSA**		
AOS1probeF1		AAGGTGGTGAGCCCAATCACCACCTTACTGGTTGACCTGCCGAAGACCGCTCCGCTGGCC
AOS1probeR1		GGCCAGCGGAGCGGTCTTCGGCAGGTCAACCAGTAAGGTGGTGATTGGGCTCACCACCTT
AOS1probeF2		AAGGTGGTGAGCCCAATCACCACCTTACTGGTTGAAATGCCGAAGACCGCTCCGCTGGCC
AOS1probeR2		GGCCAGCGGAGCGGTCTTCGGCATTTCAACCAGTAAGGTGGTGATTGGGCTCACCACCTT
AOS1probeF3		AAGGTGGTGAGCCCAATCACCACCTTACTGGTTGGGGTGCCGAAGACCGCTCCGCTGGCC
AOS1probeR3		GGCCAGCGGAGCGGTCTTCGGCACCCCAACCAGTAAGGTGGTGATTGGGCTCACCACCTT
AOS1probeF4		AAGGTGGTGAGCCCAATCACCACCTTACTGGTAAGGCTGCCGAAGACCGCTCCGCTGGCC

AOS1probeR4		GGCCAGCGGAGCGGTCTTCGGCAGCCTTACCAGTAAGGTGGTGATTGGGCTCACCACCTT
AOCprobeF		TTGGAAAGGTACGATGTCAAAAAAATAAATTTGACCATTATTTTCTATTATAATATGTAT
AOCprobeR		ATACATATTATAATAGAAAATAATGGTCAAATTTATTTTTTTGACATCGTACCTTTCCAA
LOX1probeF		TCGGTCCGATCGATCGAGTCCACGGCCATGAGCTTAGCTTGTCATGCACGTAGCTTAATTAGGCCCGGAACTTCCAGTCT
LOX1probeR		AGACTGGAAGTTCCGGGCCTAATTAAGCTACGTGCATGACAAGCTAAGCTCATGGCCGTGGACTCGATCGATCGGACCGA
**Primers for dual luciferase transcriptional activity assay**		
AOS1-XhoI-F		AATCTCGAGGGAGTACTAGCAGCTAGCAG
AOS1-SalI-R		TAAGTCGACTTCATGTCCATCTCGTGCCC
WRKY72-XbaI-F		GCTCTAGAATGGAGAACTTCCCGATACTCTTTG
WRKY72-KpnI-R		GGGGTACCCTACTGGAACATGTGGGAAGCAGCA
**Primers for ChIP-qPCR**		
cAOS1F1		ACGCACTGGGCGTAAAAG
cAOS1R1		GAGGAGCCCTAAGACACC
cAOS1F2		TCTCCTCCACTTTTAAAA
cAOS1R2		CTAGCTCCAACTGCAAGT
cAOS1F3		TTGCATCTCGTTCGCGTC
cAOS1R3		AAGTACTCGTACCTGTCC
cAOS1F4		TCTTCACCGGCACCTTCA
cAOS1R4		AGAAGAGGAGGGTCTTGA
cAOS1F5		TGCACGACAAGCAGTGCG
cAOS1R5		TCAGAAGGTGGCCTTCTTGA
**Primers for DNA bisulfite conversion**		
mAOS1F		TAGGTGTAGGTGTGTAGATGGTGTTA
mAOS1R		CTCACCTACTAACTACTAATACTCC

Transparent Methods

Plant materials

Rice cultivar Nipponbare (*Oryza sativa* ssp *japonica*) and all transgenic plants used in this study were planted in the experimental field and greenhouse in China National Rice Research Institute. Plants in booting-stage were used for artificial *Xoo* inoculation assay.

Vector construction and rice transformation

The coding sequence (CDS) of *WRKY72* was PCR amplified from Nipponbare leaf cDNAs. Mutations of the phosphosite on WRKY72 were introduced by PCR using synthesized oligos. The CDS fragments were ligated into vector pU1301 under the driving of a maize ubiquitin promoter (Zhang et al., 2010). For *OxWRKY72-FLAG*, the CDS without stop codon was in frame fused with 3X FLAG tag at the end with *Kpn*I and *Bam*HI sites and cloned into pU1301. CRISPR/Cas9 system for *WRKY72* knock-out construct was adopted from a previous report (Ma et al., 2015). Annealed double strand oligos of the gDNA sequences were cloned into the pYLgRNA-OsU3 using *Bsa*I site (Thermo, Waltham, U.S.A.). All the constructs were introduced into *Agrobacterium* strain EHA105 and then transformed into Nipponbare embryonic calli. The sequences of the primes used are presented in Table S2.

Rice bacterial blight inoculation

Virulent *Xoo* strain (ZJ173) was used for the inoculation assay. Briefly, booting stage plants were inoculated with ZJ173 (3×10^8/mL) by a leaf clipping method (Chen et al., 2002). Disease was scored as the percent lesion area (lesion length/leaf length) at 14 days after inoculation. The bacterial growth rate for ZJ173 strain was also determined by counting colony forming units (CFU).

Quantitative RT-PCR (qRT-PCR)

Total RNA of various tissues were isolated using Trizol (Invitrogen, Carlsbad, U.S.A.), and then reverse transcribed using first strand cDNA synthesis Kit (Toyobo,

Shanghai, China). qPCR was conducted using gene-specific primers and THUNDERBIRD SYBR® qPCR Mix (Toyobo, Shanghai, China) on a BioRad real-time PCR CFX96 system. An ubiquitin gene was used as an internal control. The data was analyzed by evaluating threshold cycle (CT) values. The relative mRNA level of tested genes was normalized to ubiquitin gene and calculated by the $2^{-\Delta\Delta CT}$ method. The experiment was performed with three biological replicates.

Subcellular localization analysis

The full CDS of *WRKY72* was ligated into vector pBWA(V)-HS fused to generate the *pro35S*:WRKY72-GFP construct. The rice protoplast was prepared as previously described (Qiu et al., 2016). Around 5 grams rice leaf was stripped into 0.5 mm size, which were digested in 10 mL enzyme solution (1.5% cellulose R10, 0.75% macerozyme R10, 0.6 M mannitol, 10 mM MES pH=7.5) for 6 hours in dark with gentle shaking at 28℃. The filtered protoplasts were washed with 10 mL ice cold W5 solution (154 mM NaCl, 125 mM $CaCl_2$, 2 mM KH_2PO_4, 2 mM MES, 5 mM glucose, pH 5.7) two times, and finally suspended in 500 μL MMG solution (0.4 M mannitol, 15 mM $MgCl_2$, 4 mM MES, pH=5.8). Then, the plasmid was transformed into the prepared protoplast by incubating in PEG (0.6 M mannitol, 100 mM $CaCl_2$, 40% PEG4000) for 30 minutes at room temperature. *pro35S*:D53-mKate was used as a nuclear marker. Lastly, the fluorescent protein signals were observed under a confocal microscope (Leica, Wetzlar, Germany).

Yeast two-hybrid assays

The matchmaker GAL4 two-hybrid system (Clontech, CA, U.S.A.) was used for Y2H assays. Full-length CDS of *WRKY72* was cloned into the pGADT7 vector, and the CDS of *SAPK10* was cloned into the pGBKT7 vector. Primers used are listed in Table S2. Constructs were co-transformed into the yeast strain Y2H Gold. SD plates lacking Trp and Leu were used to select the co-transformed colonies. The protein interactions were detected by the visualization of blue colonies on the SD plates with X-α-Gal (0.04 mg/mL) and Aureobasidin A (100 ng/mL), lacking Trp, Leu, and Ade.

Pull-down assays

Full-length CDS of *WRKY72* and *SAPK10* were cloned into pGEX-4T-1 (GE Healthcare, Chicago, U.S.A.) and pET28a (Thermo, Waltham, U.S.A.) vectors, respectively. Primers used are listed in Table S2. The recombinant protein GST-WRKY72 and HIS-SAPK10 were produced in *E.coli* DE3 (Transgen, Beijing, China), and purified using the GST-Sefinose ™ Kit (Sangon Biotech, Shanghai, China) and 6× HIS-Tagged Protein Purification Kit (CWBIO, Beijing, China) according to the manuals, respectively. The tested interactive proteins were incubated with glutathione high capacity magnetic agarose beads (Sigma-Aldrich) in pull-down buffer (50 mM Tris-HCl, pH 7.5, 5% glycerol, 1 mM EDTA, 1 mM DDT, 1 mM PMSF, 0.01% Nonidet P-40, and 150 mM KCl) at 4 °C for 2 hours. After washing five times with pull-down buffer, the beads were suspended in 50 μL 1 × PBS and 10 μL 6 × SDS protein loading buffer for 10% SDS-polyacrylamide (PAGE) gel electrophoresis and immunoblotting analysis. Proteins were detected using Supersignal West Pico Chemiluminescent Substrate (Thermo, Waltham, U.S.A.) and the ChemDoc™ Touch Imaging system (Bio-Rad). The dilution for anti-HIS (Cat: CW0083, CWBIO, Beijing, China) and anti-GST (Cat: CW0085, CWBIO, Beijing, China) was 1: 5000.

Co-IP assays

The full-length CDS of *WRKY72* and *SAPK10* were cloned into pF3PZPY122 (Menon et al., 2005) and pCAMBIA1300-GFP vectors, respectively. Primers used are listed in Table S2. WRKY72-FLAG was transiently co-expressed with empty GFP or SAPK10-GFP in tobacco leaves by *Agrobacterium* infiltration. Two grams of the transformed tissues were ground into fine powders and resuspended in protein extraction buffer (25 mM Tris-HCl, pH7.4, 150 mM NaCl, 1 mM EDTA, 1% NonidetP-40, 5 % glycerol, 1 mM PMSF, 20 μM MG132, and 1x Roche protease inhibitor cocktail (Roche, Basel, Switzerland). After a brief centrifugation (20,000g for 10 minutes), the resulting supernatant was incubated with anti-FLAG M2

magnetic beads (Sigma-Aldrich, St Louis, U.S.A.) at 4 °C for 2 hours. Subsequently, the beads were washed five times with washing buffer (50 mM Tris, pH 7.5, 150 mM NaCl, 0.2% Triton X-100, 1 mM PMSF, and 1x Roche protease inhibitor cocktail), eluted with 50 μL FLAG elution buffer (25 mM Tris-HCl, pH 7.5, and 0.2 mg/mL 3x Flag peptide) (Sigma-Aldrich, St Louis, U.S.A.) at 25 °C for 30 minutes. Proteins were detected using Supersignal West Pico Chemiluminescent Substrate (Thermo, Waltham, U.S.A.) and the ChemDoc™ Touch Imaging system (Bio-Rad, CA, U.S.A.). The dilution for anti-FLAG (Sigma-Aldrich, St Louis, U.S.A.) and anti-GFP (Cat: CW0086, CWBIO, Beijing, China) antibodies was 1:5000.

In *E.coli* phosphorylation assays

Different forms of *WRKY72* (*WRKY72*, $WRKY72^{S71A}$, $WRKY72^{S73A}$, $WRKY72^{T86A}$, and $WRKY72^{T129A}$) were cloned into the pGEX-4T-1 vector, respectively. The different truncated version of *GST-WRKY72* constructs was generated by overlap-PCR. Full-length CDS of *SAPK10* were cloned into pET28a vector. Primers used are listed in Table S2. GST-WRKY72 or GST tag alone were co-expressed with HIS-SAPK10 in *E. coli* strain DE3 (Transgen, Beijing, China), and purified using the GST-Sefinose ™ Kit (Sangon Biotech, Shanghai, China). The purified proteins (100 ng) were incubated with CIAP (Takara, Dalian, China) at 37 °C for 30 minutes, and then subjected to immunoblotting analysis of phosphorylated proteins using biotinylated Phos-tag™ zinc complex BTL111 purchased from Wako (http://www.Phos-tag.com).

EMSA assays

p-GST-WRKY72 (phosphorylated GST-WRKY72) were purified from the *E.coli* co-expressing GST-WRKY72 and HIS-SAPK10. Biotin labeled oligonucleotides were synthesized by TsingKe Company (seen in Table S2). Equal amount of the probe oligos was mixed, heated to 95°C for 2 minutes, and annealed by gradually cooling down to 25°C. Then, the purified proteins were pre-incubated with the binding buffer (10 mM Tris-HCl, pH 7.5, 50 mM KCl, 1 mM DTT, 2.5% glycerol, 5 mM $MgCl_2$, 50 ng/μL poly(dI-dC), and 0.05% NP-40) at room temperature for 20

minutes, followed by incubating with 20 fmol labeled probes with or without non-labeled competitive probes for another 20 minutes. Subsequently, the incubated samples were electrophoresed on 6% PAGE gels running with 0.5X Tris-borate-EDTA buffer (TBE) and transferred to a Nylon membrane at 100 V for 30 minutes and cross-linked on a transilluminator equipped with 312 nm bulbs. Lastly, the fluorescence signaling was detected by Chemiluminescence according to LightShift® Chemiluminescent EMSA Kit (Cat: 20148, Thermo, Waltham, U.S.A.).

Dual Luciferase transcriptional activity assay in rice protoplasts

The Dual Luciferase Reporter Gene Assay Kit (Beyotime, Shanghai, China) was used to measure the luciferase activity. Firstly, the transformed protoplasts were re-suspended in 50 μL Lysis buffer, and 30-50 μL of lysate was used to measure the luciferase activity in one well of 96-well plate. 100 μL of firefly luciferase assay substrate buffer was added into the lysate and the firefly luciferase (fLUC) activity was measured with the Infinite® 200 Pro (Tecan, Mannedorf, Switzerland). After that, 100 μL of Stop & renilla luciferase substrate buffer was added to the reaction and the renilla luciferase (rLUC) activity was measured. Relative luciferase activity was calculated as the ratio between fLUC and rLUC (fLUC/rLUC). 35S, 35S:GAL4-fLUC, and AtUbi:rLUC were used as an effector, a reporter and an internal control, respectively. Triple biological repeats were performed for each sample. Primers used for the vector construction are listed in Table S2. All of the plasmids used in this assay were purified with the Plasmid Midi Kit (Qiagen, Dusseldorf, Germany).

ChIP-quantitative PCR (ChIP-qPCR)

ChIP was performed as described previously (Hou et al., 2015). Briefly, chromatin was isolated from 2 g cross-linked leaves of *proUbi:WRKY72-FLAG* plant. Isolated chromatin was sheared to approximately 100 to 500 bp by sonication. Then, the DNA/protein complex was immune-precipitated with ChIP-grade antibody against FLAG (Cat: F1804, Sigma-Aldrich, St Louis, U.S.A.). After reverse cross-linking and proteinase K treatment, the immunoprecipitated DNA was extracted with

phenol/chloroform. The immunoprecipitated and input DNA was used for quantitative PCR using gene specific primers (seen in Table S2). The quantitative PCR results were analyzed by following a method reported in the manual of Magna ChIP™ HiSens kit (Millipore, MA, U.S.A.). All the quantitative ChIP-PCR was performed in three biological replicates. The enrichment values were normalized to the input sample.

Quantification of hormones

Free JA quantification was conducted and the methods were modified form a previous report using the Waters ACQUITY UPLC Xevo TQ HPLC-MS/MS system (You et al., 2016). Approximately 0.5 g leaf samples were finely ground in liquid nitrogen and extracted with 10 mL buffer (isopropanol: water: hydrochloric acid, 2: 1: 0.002 v/v). The extracts were shaken at 4°C for 30 minutes and 20 mL dichloromethane was then added. After that, the samples were shaken at 4°C for 30 minutes and centrifuged at 13,000 rpm for 5 minutes. The organic phase was extracted and dried under liquid nitrogen. The pellets were dissolved in 150 μL methanol (0.1% methane acid) and filtered with a 0.22 μm filter membrane. Lastly, the purified product (2 μL each injection) was subjected to HPLC-MS/MS analysis. The quantitative data was obtained using the peaks of the precursor ions 209.2 and the product ions 58.9. MS conditions were as follows: the spray voltage was 4,500 V; the pressure of the air curtain, nebulizer, and aux gas were 15, 65, and 70 psi, respectively; and the atomizing temperature was 400°C. The experiment was performed with three biological replicates.

DNA bisulfite conversion for Sanger sequencing and methylation analysis

Total genomic DNA was extracted using a DNeasy Plant Maxi Kit (Qiagen, Dusseldorf, Germany) and modified using an EpiTect Bisulfite kit (Qiagen, Dusseldorf, Germany) according to the manufacturer's instructions. Then, the modified DNA was amplified, purified and cloned into pMD18-T vector (Takara, Dalian, China). The amplified primers were designed online

(http://www.urogene.org/methprimer/) and seen in Table S2. Lastly, At least 30 clones were sequenced for each sample and the bisulfite sequencing results were analyzed by the Kismeth website (http://katahdin.mssm.edu/kismeth). The methylation levels of the three types of cytosines (CG, CHG, CHH) were calculated by dividing the number of non-converted (methylated) cytosines by the total number of cytosines.

References

Ma, X., Zhang, Q., Zhu, Q., Liu, W., Chen, Y., Qiu, R., Wang, B., Yang, Z., Li, H., and Liu, Y. (2015). A robust CRISPR/Cas9 system for convenient, high-efficiency multiplex genome editing in monocot and dicot plants. Mol Plant ***8***, 1274-1284.

Menon, S., Rubio, V., Wang, X., Deng, X.W., and Wei, N. (2005). Purification of the COP9 signalosome from porcine spleen, human cell lines, and Arabidopsis thaliana plants. Methods Enzymol ***398***, 468-481.

Qiu, J., Hou, Y., Tong, X., Wang, Y., Lin, H., Liu, Q., Zhang, W., Li, Z., Nallamilli, B.R. and Zhang, J. (2016) Quantitative phosphoproteomic analysis of early seed development in rice (Oryza sativa L.). *Plant Mol Biol* ***90***, 249-265.

You, C., Zhu, H., Xu, B., Huang, W., Wang, S., Ding, Y., Liu, Z., Li, G., Chen, L. and Ding, C. (2016) Effect of Removing Superior Spikelets on Grain Filling of Inferior Spikelets in Rice. *Frontiers in Plant Science* ***7***, 1161.

Zhang, J., Nallamilli, B.R., Mujahid, H., and Peng, Z. (2010). OsMADS6 plays an essential role in endosperm nutrient accumulation and is subject to epigenetic regulation in rice (Oryza sativa). Plant Journal ***64***, 604–617.
